# Clinico-pathological features and mutational spectrum of 16 nemaline myopathy patients from a Chinese neuromuscular center

**DOI:** 10.1007/s13760-020-01542-9

**Published:** 2021-03-19

**Authors:** Xi Yin, Chuanqiang Pu, Zhenfu Wang, Ke Li, HuiFang Wang

**Affiliations:** 1 Department of Neurology, The Second Medical Center and National Clinical Research Center for Geriatric Disease, Chinese PLA General Hospital, 28 Fuxing Road, Beijing, 100853 China; 2 Department of Neurology, The First Medical Center, Chinese PLA General Hospital, 28 Fuxing Road, Beijing, 100853 China; 3 Department of Neurology, the First Hospital of Shanxi Medical University, 85 Jiefang South Road, Taiyuan, 030001 China

**Keywords:** Nemaline myopathy, Clinical features, Pathology, Gene mutation

## Abstract

Nemaline myopathy (NM) is a congenital myopathy of great heterogeneity, characterized by the presence of rods in the cytoplasm of muscle fibers. The samples of 16 nemaline myopathy patients diagnosed by characteristically pathological features went through whole exon sequencing. Clinico-pathological and genetic features of the cases were systematically analyzed. According to the classification of nemaline myopathy by ENMC, 8 cases are typical congenital subtype, 6 cases are childhood/juvenile onset subtype and 2 case are adult onset subtype. In histological findings, characteristic purple-colored rods are discovered under modified gömöri trichrome staining (MGT). Electron microscopy revealed the presence of high electron-dense nemaline bodies around the submucosa and the nucleus nine patients (9/16 56.3%) were detected pathogenic causative mutations, among whom mutations in the *NEB* gene were the most frequent (6 patients, 66.7%). *KBTBD13* gene mutation was discovered in two patients and *ACTA1* gene mutation was discovered in 1 patient. Nemaline myopathy is a congenital myopathy with highly clinico-pathological and genetic heterogeneity. *NEB* gene mutation is the most common mutation, in which splicing change c.21522 +3A > G is hotspot mutation in Chinese NM patients.

**Supplementary Information:**

The online version contains supplementary material available at 10.1007/s13760-020-01542-9.

## Introduction

Nemaline myopathy (NM) is a common type of congenital myopathy, which is named by the presence of rod-like structures in skeletal muscle fibers [1, 2]. In 2000, European Neuromuscular Center (ENMC) International Consortium defined six types of NM according to different clinical manifestations [3]: severe, intermediate, typical congenital, childhood/juvenile onset, adult onset and other unusual forms. In nemaline myopathy, various genetic mutations cause structural component changes of skeletal muscle, leading to hypotonia or general weakness predominantly affecting facial, axial, and proximal limb muscles. Until now, 13 mutant genes have been discovered [4–13]: *TPM3, NEB, ACTA1, TPM2, TNNT1, CFL2, KBTBD13, KLHL40, KLHL41, LMOD3, MYPN, MYO18B, TNNT3*. In our previous study, we have systematically reviewed the clinical and pathological features of nemaline myopathy patients in China [14]. But there is no systemic research on the genetic features of nemaline myopathy patients in China until now. Here, in order to investigate the clinical features and mutational spectrum of nemaline myopathy in China, we present 16 nemaline myopathy cases from our neuromuscular disease database with clinico-pathological and genetic data.

## Patients and methods

### Patients

A total of 16 patients were included in the study. From April 1986 to October 2018, 5429 muscle biopsies were performed on patients with suspected myopathy in our department, and 16 patients (0.29%) were diagnosed nemaline myopathy according to characteristic pathological features. Written informed consent was obtained from each patient. The study was approved by the institutional Review Board of Chinese PLA General Hospital.

### Clinical information and Histological analysis

Detailed clinical data were collected from each patient, which included detailed personal history (age at onset, initial symptom or sign, motor milestone delay, clinical course and family history); clinical features (distribution of muscle weakness and atrophy, progression pattern, dysphagia and mental retardation); dysmorphic features (pseudo hypertrophy, high arched palate and foot, elongated face and spinal deformity); and muscle power (manual muscle testing by physicians).

Blood samples of the nemaline myopathy patients were also collected and the level of serum creatine kinase (CK) was test by ELISA according to manufacturer’s protocols (Sinopharm Chemical Reagent, Beijing, China). For electromyography and nerve conduction velocity tests, different muscle parts of patients including bilateral deltoid muscle, biceps brachii muscle, quadriceps femoris muscle and anterior tibial muscle were tested by the keypoint 2000 equipment (Dantec Dynamics, Skovlunde, Denmark). After inserting electrode into muscles, the neurologist recorded signals form the muscle at rest and contraction. The nerve velocity was measured by the distance and the time that the signal transmitted along nerves which is recorded by keypoint 2000.

Open muscle biopsy was performed on the clinically affected limb muscles (biceps brachii or quadriceps femoris mostly) under local anesthesia. Following removal, muscle samples were immediately frozen in isopentane cooled with liquid nitrogen and were stored at − 80 °̊C. Transversal serial 5-μm frozen muscle sections were stained with hematoxylin and eosin staining (HE), modified gömöri trichrome staining (MGT), reduced nicotinamide adenine dinucleotide dehydrogenase-tetrazolium reductase staining (NADH-TR). Morphometric evaluation of the muscle specimens was performed under a light microscope (B × 51, Olympus, Tokyo, Japan). 0.5 × 0.5 × 1.0 cm-size muscle sample fixed in 2% glutaraldehyde, post fixed with 1% osmium tetroxide, was embedded in araldite. Ultrathin sections were stained with uranyl acetate and lead citrate and were viewed and photographed with a JEM1230 electron microscope (JEOL, Tokyo, Japan). All reagents were purchased from Sinopharm Chemical Reagent Co Ltd. (Beijing, China).

### DNA analysis

Genomic DNA was extracted from peripheral blood or frozen muscle of the patients. First, all coding exons and exon–intron boundaries of *ACTA1* and *TPM3* were amplified by PCR and analyzed by Sanger sequencing. If no pathogenic mutation was identified in these two genes, other causative genes of congenital myopathies, including NM-related genes (*NEB, CFL2, TPM2, TNNT1, KBTBD13, KLHL40, KLHL41, LMOD3, MYO18B, and MYPN*), were evaluated by whole exon sequencing (WES). Exon capture was performed using the Illumina TruSeq Exon Enrichment Guide. Sequence reads were mapped to the human reference genome assembly (GRch37/hg19) using Integrative Genomics Viewer (IGV; Broad Institute, Cambridge, MA, USA). Variants potentially affecting protein structure or function, including nonsynonymous variants, frameshifts, or variants affecting splicing, were investigated. The variants showed a depth of more than 50 and a minor allele frequency of less than 0.01. We evaluated the impact of mutations using the SIFT/PROVEAN and Polyphen2 prediction system in the case of point mutations. The splice sites were evaluated with Human Splicing Finder. Sanger sequencing was performed to confirm the mutations found by WES. Because WES has clear limitations in reading the triplicate region of *NEB*, Sanger fill-in was performed for patients in whom a single heterozygous variant or no variant was identified. In addition, all patient samples were done CNV analysis by targeted array-CGH, as previously described [15].

## Results

### Clinical features

Details of the patients were shown in Table 1.

**Table 1:** Summary of clinicopathological and genetic features of 16 nemaline myopathy patients

Patient no.	1	2	3		4	5	6
Subtype	Adult onset	Typical congenial	Childhood/ juvenile onset		Typical congenital	Typical congenital	Childhood/ juvenile onset
Gender/age (at biopsy)	M/49	F/10	F/21		M/6	F/35	M/22
Family history	No	No	No		No	No	No
Age at onset	45	Birth	17		Birth	Birth	16
Mode of onset	Subacute	Chronic	Chronic		Chronic	Chronic	Chronic
Course of disease	4 years	10 years	4 years		6 years	35 years	6 years
Initial symptoms	L/Ex weakness dyspnea	L/EX weakness	L/EX weakness		L/EX weakness	Infantile hypotonia EX weakness	L/EX weakness Slow movement
Muscle atrophy	Trunk,proximal extremity	No	Gastrocnemius muscle		No	No	Proximal extremity
Dysmorphic features	No	No	No		High arched feet	No	
Clinical progression	Rapidly progressive	No progressive	Slowly progressive		No progressive	No progressive	Slowly progressive
CK(IU/l)	107.6	32	56.5		195.1	194.1	235
NCV/EMG	Normal/myopathic	Normal/myopathic	Normal/myopathic		Normal/myopathic	Normal/myopathic	Normal/myopathic
Mutant Gene			NEB				KBTBD13
Chromosome location			2				15
Hereditary type			AR				AD
Nucleotide change			c.23122-1G>C (splice change)	c.21522+3A>G (splice change)			c.1169A>C (missense change)
Predicted amino acid change							p.K390N

Patient no.	7		8	9	10	11	12
Subtype	Typical congenital		Typical congenital	Childhood/ juvenile onset	Childhood/ juvenile onset	Typical congenital	Typical congenital
Gender/age (at biopsy)	M/27		M/31	M/21	F/40	M/18	F/33
Family history	No		No	No	Son has similar symptoms	No	No
Age at onset	Birth		Birth	18	8	Birth	Birth
Mode of onset	Chronic		Chronic	Chronic	Chronic	Chronic	Chronic
Course of disease	27 years		31 years	4 years	6 years	18 years	33 years
Initial symptoms	L/EX weakness		EX weakness	L/EX weakness	EX weakness slow movement	Delayed motor milestone L/EX weakness	Delayed motor milestone L/EX weakness
Muscle atrophy	L/EX, severe in distal		Four extremities	No	No	L/EX, severe in distal	L/EX,severe in distal
Dysmorphic features	High arched feet			High arched feet			
Clinical progression	Slowly progressive		Slowly progressive	Slowly progressive	Slowly progressive	Slowly progressive	Slowly progressive
CK(IU/l)	110.4		69	113.2	78	99.8	96.3
NCV/EMG	Normal/myopathic		Normal/myopathic	Normal/myopathic	Normal/myopathic	Normal/myopathic	Normal/myopathic+neurogenic
Gene	NEB		ACTA1		KBTBD13	NEB	
Chromosome location	2		1		15	2	
Hereditary type	AR		AD		AD	AR	
Nucleotide change	c.14837dupA (shift change)	c.3758C>A (missense change)	c.956T>C (missense change)		c.1170G>C (missense change)	c.21522+3A>G (splice change) c.11164C>T (nonsense change)	
Predicted amino acid change	p.Q4947Afs*12	p.T1253K	p.I319T		p.K390N	p.R3722*	

Patient no.	13		14		15	16	
Subtype	Typical congenital		Childhood/ juvenile onset		Childhood/ juvenile onset	Adult onset	
Gender/age (at biopsy)	M/24		M/21		M/35	F/30	
Family history	Brother had similar symptoms		No		No	Brother had similar symptoms	
Age at onset	Birth		18 years old		20 years old	25 years old	
Mode of onset	Chronic		Chronic		Chronic	Chronic	
Course of disease	24 years		3 years		15years	5 years	
Initial symptoms	Delayed motor milestone, dysphagia		L/EX weakness		L/EX weakness	L/EX weakness	
Muscle atrophy	Facial muscle, sternocleidomastoid, L/EX		No		No	No	
Dysmorphic features							
Clinical progression	Progressive		Slowly progressive		Slowly progressive	Progressive	
CK(IU/l)	64.1		83.7		62.9	64.8	
NCV/EMG	Normal/myopathic		Normal/myopathic		Normal/myopathic	Normal/myopathic	
Gene	NEB		NEB			NEB	
Chromosome location	2		2			2	
Hereditary type	AR		AR			AR	
Nucleotide change	c.21522+3A>G (splice change)	c.4417C>T (nonsense change)	c.21522+3A>G (splice change)	c.23233-1G>T (splice change)		c.21522+3A>G (splice change)	c.5343+1G>A (splice change)
Predicted amino acid change		p.R1473*					

*M* male; *F* female; *L/Ex* lower extremity; *Ex* extremity; *CK* creatine kinase; *NCV* nerve conduction velocity; *EMG* electromyograpy; *AR* autosomal recessive; *AD* autosomal dominant

From January 1986 to October 2018, 5,429 suspected myopathy cases received muscle biopsies in our center. The proportion of NM cases in this group was 0.29% (16/5,429). In the 16 patients evaluated in this study, there were ten males (M) and six females (F), with a ratio M:F = 1:0.6. The patients’ age ranged from 6 to 49 years, with a mean age 26.7 ± 10.8 years. The disease course ranged from 3 to 35 years, with a mean disease course of 14.4 ± 11.8 years.

According to the classification of nemaline myopathy by European Neuromuscular Center (ENMC) in 2000, 16 patients in our study were divided into three groups: 8 cases belong to typical congenital subtype, 6 cases belong to childhood/juvenile onset subtype and 2 cases belong to adult onset subtype. Patients of typical congenital subtype presented hypotonia and chronic limb weakness during the neonatal period, with delayed motor milestone. The patients of childhood/juvenile onset subtype usually show lower limbs weakness without muscle atrophy as the initial manifestation. While with the progression of disease, lower limbs muscle atrophy will gradually appear. The course of the case with adult onset subtype progressed rapidly, the patient developed trunk and proximal extremities muscle atrophy in several years. Among all the patients, the creatine kinase was normal or slightly elevated, with an average of 103.9 ± 56.8 IU/L (range 32–235 IU/L). EMG results suggested myogenic damage and normal nerve conduction velocity.

### Pathological features

**Fig. 1:**
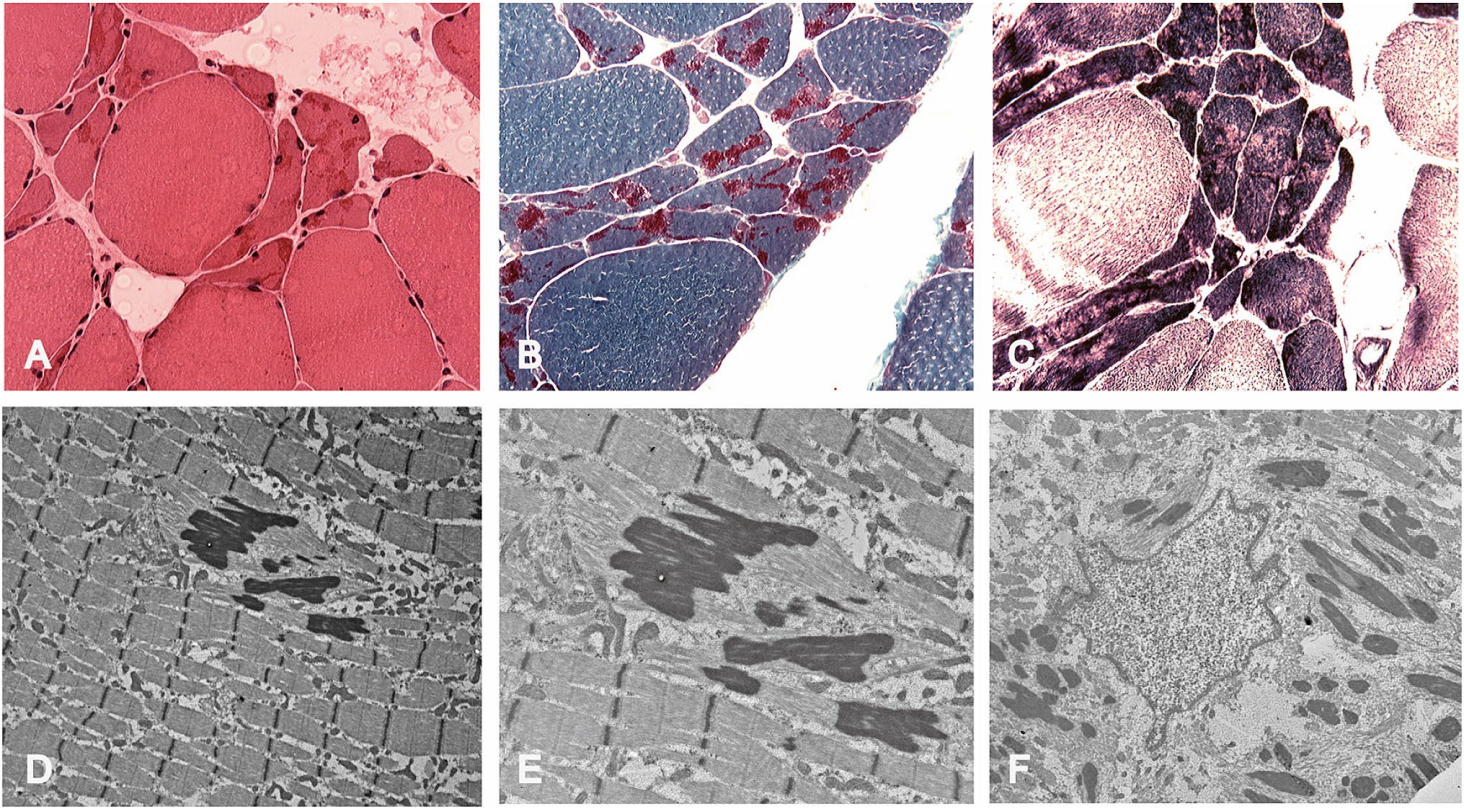
Histochemical, Ultrastructural findings in the muscle biopsy samples from nemaline myopathy patients (Case 3). HE staining indicates that collection of rods of eosinophilic rods assemble under the sarcolemma of many muscle fiber membranes. (X400, **a**); MGT staining suggests that typical purple-colored rods are observed in the subsarcolemmal region of muscle fibers. (X400, **b**); NADH-TR staining shows areas deviod of oxidative enzyme activity in which rods exist. (X400, **c**); Electron microscopy show rod-like structure with high electron density among myofibrils. (X8000, **d**); The rod-like structure with high electron density among segments of myofibrils, and the surrounding myofibrillar structure is ruptured. (X15000, **e** Enlarged figure of **d**); Electron microscopy show that there are many rod-like structures with different electron densities near the nucleus (X10000, **f**)

As shown in Fig. 1, HE staining revealed variations in the muscle fiber size, with areas of small groups of atrophic muscle fibers. No muscle fiber necrosis and no inflammatory cell infiltration were discovered. Collection of eosinophilic rods assembled under the sarcolemma. Typical purple-colored rods were observed in the subsarcolemmal region of muscle fibers stained with MGT staining. NADH-TR staining could not show the rods, while the light stained area without oxidative enzyme activity usually means collections of rods. Electron microscopy showed that some myofibrillar dysplasia was manifested as myofilament rupture, irregular alignment and different myofibrils. The Z-line of myofibrils was small, irregular or disappeared. There are many uniform high electron density rods around the submucosa and the nucleus. Most rods were arranged equally with myofibrils, even a segment of myofibrils.

### Genetic analysis results

All 16 patients were underwent genetic analysis and nine patients (9/16 56.3%) were detected gene mutations. Among them, *NEB* gene mutation was discovered in six patients, *KBTBD13* gene mutation was discovered in two patients and *ACTA1* gene mutation was discovered in one patient. For the six patients harboring *NEB* gene mutation, we discovered the splice change c.21522 + 3A > G in five patients, which have been reported before [15]. And one nonsense change c.4417C > T and two splice changes c.23233-1G > T/c.5343 + 1G > A were newly discovered nucleotide changes according HGMD database for *NEB* gene in nemaline myopathy patients. For the two patients harboring *KBTBD13* gene mutation, two missense mutations were discovered, the c.1170G > C (p.K390N) mutation was reported before [7] and the c.1169G > C (p.K390N) was novel mutation. For one patient harboring ACTA1 gene mutation, the missense mutation c.956 T > C (p.I319T) was a novel one which has not been reported before.

## Discussion

Nemaline myopathy is one type of congenital myopathy, the cause of which is associated with multiple gene mutations. Our study systematically analyze the clinical and pathological characteristics of nemaline myopathy. And importantly, the genetic features of 16 cases of nemaline myopathy collected in our center were studies by WES, in which nine mutations were discovered, which broaden gene mutation spectrum of nemaline myopathy in China.

According to our data, nemaline myopathy is a rare neuromuscular disease, which accounts for 0.29% in all the 5429 patients of our myopathy database. The clinical presentation of NM patients is greatly variable, ranging from neonatal death to mild limb weakness. The muscle weakness of nemaline myopathy is usually generalized, with involvement of the neck flexors, the face and proximal muscles. Traditionally, proximal muscle weakness is characteristic in nemaline myopathy, while some patients suffer from distal muscle weakness, which suggests the heterogeneity of clinical manifestation and brings challenge for the diagnosis of nemaline myopathy. Case 7 and 11 in our study showed distal muscle weakness with *NEB* gene mutations, which is in accordance with the study by Kiiski, who reported distal nemaline/cap myopathy with a large deletion in the nebulin gene [16]. According to the ENMC classification, the 16 patients in our study were divided into three groups: typical congenital, childhood/juvenile onset, adult onset, and the proportion is 50%, 37.5%, 12.5% respectively, which is similar to the previous research [17]. There are two adult onset cases (1 and 16) with different disease progression. Case 1 in our study is sporadic late-onset nemaline myopathy (SLONM) subtype, which is usually immune-mediated and related to monoclonal gammopathy of undermined significance (MGUS) [18, 19]. He showed rapidly progressive disease course with limbs and trunk weakness [20]. Without approriate treatment, the prognosis of SLONM is unfavorable and the patients might suffer from progressive respiratory failure. Previous studies have suggested that early monitoring and appropriate treatment of respiratory function for NM patients could effectively improve their quality of life and prolong survival. Case 16 had the age of onset at 25 years old, while he had family history and harbored *NEB* gene mutation. We assume that the reason for his onset so late is due to the chronic progression of the disease, and he did not show symptoms until the age of 25. With the deepening understanding of the NM, in 2019, Sewry and his colleagues proposed a new classification for memaline myopathies which gave a strong genotype–phenotype correlation, which is a great progression with the development of gene detection technique [21]. According to the new classification, cases in our study could be divided into three groups: congenital nemaline myopathy (Case 3, 7, 8, 11, 13, 14), mild (childhood or juvenile onset) nemaline myopathy (Case 16) and Childhood-onset nemaline myopathy with slowness of movements and core-rod histology (Case 5, 10).

With great clinical heterogeneity of nemaline myopathy, the diagnosis of NM is based on the appearance of dark red staining inclusions with the MGT stainning in muscle pathology [2]. While HE stained sections of skeletal muscle from patients with NM can appear normal, or exhibit some fiber size variation. By electron microscopy, rods are electron dense ovoid structures which are often parallel to the long axis of the sarcomere or near the Z disc, measuring 1–7 µm in length and 0.3–2 µm in width. Luther et al. [22] have demonstrated that rods have a lattice structure similar to Z-disks on electron microscope and the major constituent of rods is α-actinin, which is exactly the component of Z disc.

As congenital myopathy, nemaline myopathy is mainly caused by gene mutations which is related to different components of striated muscle. With the development of gene sequencing technology, 13 genes have been discovered to be associated to the onset of nemaline myopathy. Among these, *NEB* gene mutations are responsible for 50% of the cases of NM [23]. *NEB* gene mutations are seen most commonly in autosomal recessive NM cases and these patients present as typical congenital NM [24]. *NEB* gene is a large one which contains 183 exons in a 249 kb genomic region. It encodes a 600 to 900 kD protein nebuline which is one component of thin filaments within the sarcomeres of skeletal muscle and function as molecular ruler in the regulation of muscle contraction, Z-disc formation, myofibril organization and assembly [25]. Kiss and colleagues discovered shorter thin filament as well as impaired tropomyosin and troponin movement in nebulin-knockout mice, which indicates that nebulin participate the thin filament activation and cross-bridge recruitment [26]. In our study, 67% patients harbor *NEB* gene mutations. And all the patients with *NEB* gene mutations showed relatively benign clinical course with slow clinical progression. Splicing change c.21522 + 3A > G has been reported before and the research reported only one case [15]. While in our research, 5 out 6 unrelated nemaline myopathy patients with *NEB* mutations harbored this mutational site, which reminded us that this mutational site might be a mutational hotspot for nemaline myopathy patients in China. And there were three *NEB* mutations firstly discovered in our study including one nonsense change c.4417C > T and two splicing change c.23233-1G > T/c.5343 + 1G > A which give rise to truncated nebulin without normal function and results in reduced cross-bridge interaction and impaired muscle force.

Except *NEB* gene mutation, *ACTA1* gene mutation is the most common one that cause nemaline myopathy. The *ACTA1* gene encodes skeletal muscle α-actin, the principal actin isoform in adult skeletal muscle, which is highly conserved and forms the core of the thin filament of the sarcomere. Sarcomere contractility of the patients with *ACTA1* mutations reduces due to the decreased myosin heads binding to actin, which is mainly composed of α-actin encoded by ACTA1 gene [27]. The clinical manifestations of nemaline myopathy caused by *ACTA1* gene mutations is often severe, with the neonate presenting as a floppy infant, sometimes with failure to establish respiration and spontaneous movements. More rarely, mutations in *ACTA1* may cause the intermediate, mild, or typical forms of nemaline myopathy [28]. The clinical subtype of the case in our research is typical congenital, which is a rare one. The missense change c.956 T > C in highly conserved *ACTA1* gene causes disrupted protein structure and finally impaired muscle contraction.

*KBTBD13*, unlike other mutant genes encoding the thin filament component, which encodes Kelch-like proteins associated with thin filament regulation. The protein it encodes has an N-terminal BTB/POZ domain, followed by a central α-helical linker region and a C-terminal Kelch repeat domain that contains five repeats and is predicted to form a β-propeller structure. Mutations in *KBTBD13* gene were found to cause dominantly inherited nemaline myopathy, which features onset of weakness in early childhood and the presence of both nemaline bodies and core-like formations in the muscle fibers. Cullin-3-RING ubiquitin ligase mediates the proteostasis in skeletal muscle and acts as critical regulator in the muscle development and function. As the E3 ubiquitin ligase Cullin-3 substrate adaptors, BTB/Kelch domain proteins encoded by *KBTBD13* participate in the degradation of non-muscle α-actinins (ACTN1) during myogenesis [29, 30]. The newly discovered missense change c.1169A > C in KBTBD13 gene is located in the highly conserved fifth repeats of Kelch domain, is predicted to damage the corresponding strands of the molecular β-propeller blades of KBTBD13 protein. Then the accumulation of ACTN1 in muscle fibers will interfere the development, function and maintenance of skeletal muscle and finally give rise to reduced muscle force.

It is worth noting that nemaline myopathy cases with KBTBD13 gene mutations are rarely reported, while the muscle pathology shows characteristic rods and cores lesions [7, 31]. The characteristic pathological features suggest genotype–phenotype correlation, which has certain hints for genetic detection. In fact, it should be noted that not every patient’s pathological manifestations are typical rods and cores, which may manifest as an unevenness of stain resembling core-like areas and need to be confirmed by electron microscopy.

## Conclusion

In our study, we systemically reviewed the clinical, pathological and genetic characteristics of nemaline myopathy patients in China. The *NEB*, *KBTBD13*, and *ACTA1* gene mutations were discovered in 9 out of 16 patients in our database. Mutational hotspot c.21522 + 3A > G in *NEB* gene was discovered in nemaline myopathy patients in China and three mutational sites in *NEB* gene were reported which haven't been discovered before. The clinical and pathological characteristics of nemaline myopathy patients in China was systematically reviewed and gene mutation spectrum was broadened. Unfortunately, seven patients in our study who underwent genetic analysis had no mutation been discovered in known mutational genes related to nemaline myopathy. This reminded us that there might be undiscovered mutational genes in the cases and nemaline myopathy is a disease with great genetic heterogeneity. With the development of genetic sequencing technology, more and more mutational genes may be discovered and the pathogenesis of nemaline myopathy may be illustrated clearly.

## Supplementary Information

Below is the link to the electronic supplementary material.

Supplementary file1 (tif 10400 KB)
