# Supplementary figures and images for: Clinico-pathological features and mutational spectrum of 16 nemaline myopathy patients from a Chinese neuromuscular center
Source: Acta Neurol Belg. 2021 Mar 19;122(3):631–9. doi: 10.1007/s13760-020-01542-9 (PMC9170660; doi:10.1007/s13760-020-01542-9)

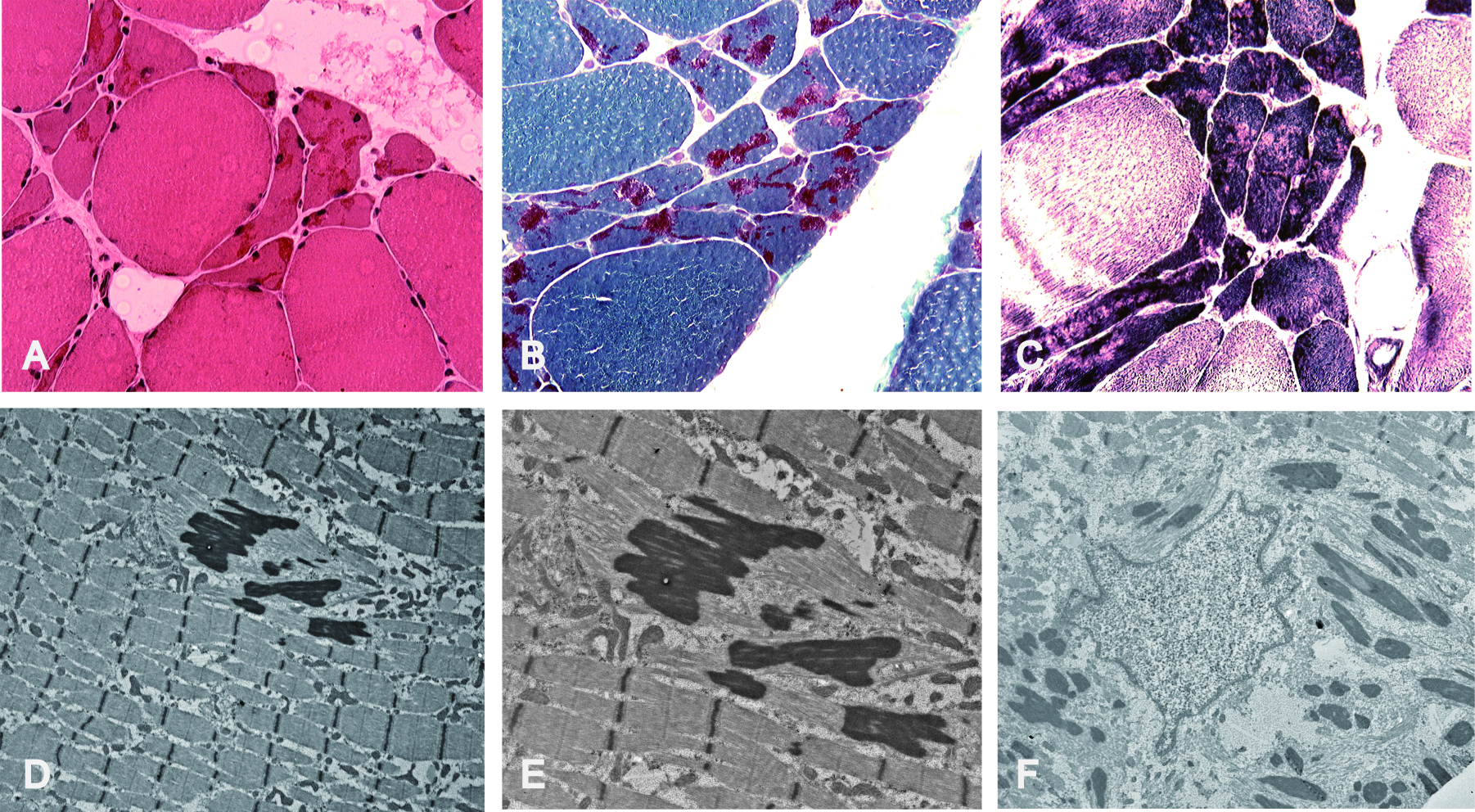

Supplement: Supplementary file 1 — Supplementary file1 (tif 10400 KB) [file 13760_2020_1542_MOESM1_ESM.tif]
